# Conjunctival melanosis associated with occupational exposure to airborne organic compounds: a case report and review of literature

**DOI:** 10.3389/fmed.2026.1945263

**Published:** 2026-09-16

**Authors:** Shuqin Xu, Chongyang Mou, Shuo Yang, Gang Wu

**Affiliations:** 1 The Second People’s Hospital of Quzhou, The Second Affiliated Hospital of Zhejiang University School of Medicine, Quzhou, China; 2 Eye Center, School of Medicine, Second Affiliated Hospital, Zhejiang University, Hangzhou, China

**Keywords:** amniotic membrane transplantation, conjunctival melanosis, conjunctivectomy, occupational exposure, organic compound

## Abstract

Conjunctival pigmentation is a multifactorial condition that may be associated with occupational exposure to airborne organic compounds, but clinical reports of such cases remain limited. A 40-year-old Han Chinese male presented with scattered black deposits on the conjunctiva and lacrimal caruncle after prolonged occupational exposure to airborne organic compounds (phenylenediamine) without personal protective equipment. He was advised to avoid further exposure. At the 3-month follow-up, the pigmentation in both eyes had partially improved. The patient requested surgical treatment for cosmetic concerns in one eye; selective excision of the pigmented conjunctiva combined with amniotic membrane transplantation was performed, achieving satisfactory outcomes. The contralateral eye was kept under observation, and after 1 year of continued avoidance of exposure, the pigmentation in the unoperated eye had further decreased substantially. Histopathology of the excised tissue revealed increased melanocytes within the conjunctival epithelial basal layer without atypia, confirming a benign reactive process and excluding malignancy. Occupationally acquired conjunctival pigmentation may partially and progressively resolve after removal of the causative agent. For patients with persistent lesions or cosmetic requests, partial conjunctival resection combined with amniotic membrane transplantation can be an effective treatment option.

## Introduction

Conjunctival pigmentation is a common clinical condition that can be caused by endogenous and exogenous factors. Environmental pollution or radiation accounts for a relatively large proportion of exogenous causes (1, 2). Exposure to air pollutants such as PM2.5 has pathogenic effects on ocular surface diseases, including dry eye, meibomian gland dysfunction, conjunctivitis, and blepharitis (3). As comprehensively reviewed by Christy et al. (4), pigmented conjunctival lesions range from incidental pigment deposition to malignant melanoma, with overlapping clinical features that require careful differentiation. In recent years, despite the identification of exogenous factors such as occupational airborne exposure, there have been few updated reports on relevant cases, outcomes after avoiding exposure sources, follow-up observations, or surgical decisions.

We recently treated a case of conjunctival pigmentation associated with occupational exposure to an airborne organic compound (phenylenediamine). Based on the clinical presentation, we first recommended that the patient immediately avoid further exposure to the contaminated substance and undergo regular follow-up. After 3 months of follow-up, the degree of conjunctival pigmentation in both eyes had reduced. To meet the patient‘s cosmetic concerns at that time, we carefully evaluated the surgical indications and therefore performed conjunctival pigmentation excision combined with amniotic membrane transplantation in one eye—a procedure performed primarily for aesthetic purposes while also providing tissue for histopathological diagnosis. Postoperative pathological examination confirmed melanocytic pigmentation and excluded malignant changes. During follow-up, the appearance of the operated eye continued to improve, while the contralateral (unoperated) eye also showed gradual reduction of pigmentation over time.

## Case presentation

A 40-year-old Han Chinese male presented with progressive darkening of the conjunctiva in both eyes over a three-month period. Slit-lamp examination revealed numerous scattered flat, non-elevated deposits in the bulbar conjunctiva, predominantly on the nasal and temporal sides, as well as in the lacrimal caruncle regions, without limbal or corneal involvement. The lesions were multifocal and discrete, with no vascularization or cysts observed (Figure 1). Visual acuity was normal (20/20 bilaterally), with no significant abnormalities observed in the cornea, lens, or fundus. The patient denied any history of ocular trauma, foreign body contact, or direct exposure to chemical substances. He had no history of any oral or topical medications prior to presentation, and no history of topical prostaglandin analogue use. He reported no previous systemic diseases. Family history was unremarkable for cutaneous or ocular pigmentation disorders. Physical examination was unremarkable except for newly detected hypertension (198/107 mmHg), which was identified at this presentation and had not been previously diagnosed or treated. We recommended that he consult the internal medicine department for further evaluation and blood pressure control. Further detailed occupational history revealed that the patient had worked in the storage warehouse for phenylenediamine powder at a dye intermediate manufacturing plant for the past 12 months, with approximately three hours of daily exposure without any personal protective equipment (PPE)—no respirator, goggles, or gloves—during work. The warehouse was a small, enclosed space; the patient was the sole worker in this storage area and worked there for prolonged periods without protection. The exposure was primarily to phenylenediamine dust generated during handling and bagging operations. No other workers were employed in the same storage area, and thus no comparison with colleagues regarding similar ocular findings could be made. Given the diagnosis of occupationally related conjunctival pigmentation, the patient was advised to immediately cease exposure and completely leave the work environment, which he did after the initial diagnosis. A clinical timeline is presented in Table 1 to facilitate understanding of the disease course.

**Figure 1:**
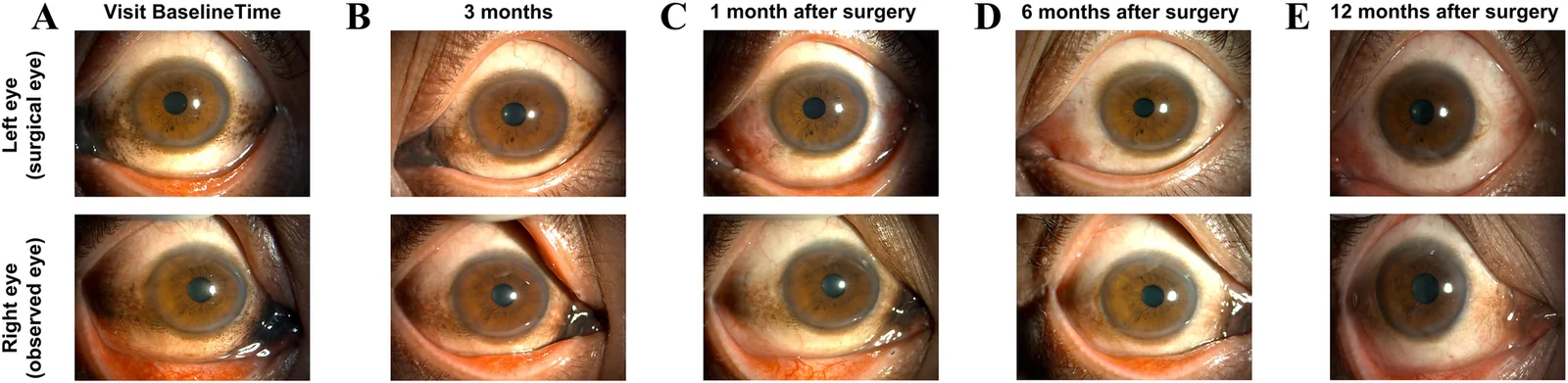
Clinical photographs of both eyes at different time points. **(A)** Baseline (initial visit): bilateral conjunctival pigmentation, with left eye (surgical eye) and right eye (observed eye) showing similar severity. **(B)** After 3 months of avoidance of exposure (preoperative): partial reduction of pigmentation in both eyes. **(C)** Postoperative 1 month (left eye after conjunctivectomy + amniotic membrane transplantation): the operated left eye shows healing conjunctiva with residual pigmentation; the unoperated right eye remains under observation. **(D)** Postoperative 6 months: the operated left eye demonstrates marked aesthetic improvement; the right eye shows substantial reduction of pigmentation. **(E)** Postoperative 12 months: the operated left eye achieves satisfactory cosmetic outcome; the unoperated right eye, after 15 months of total avoidance of exposure, exhibits substantially decreased pigmentation compared to baseline. The left eye was the surgical eye (underwent conjunctivectomy + AMT), and the right eye was the untreated fellow eye.

**Table 1:** Clinical timeline of the case.

Time Point	Event
Month-12	Started working in phenylenediamine storage warehouse (sole worker, no PPE)
Month 0 (initial visit)	Presented with bilateral conjunctival pigmentation; newly diagnosed hypertension (198/107 mmHg); advised to cease exposure and referred for hypertension management
Month +3	Partial reduction of pigmentation; left eye surgery (conjunctivectomy + AMT)
Month +4	Postoperative 1-month follow-up: graft healing, moderate aesthetic improvement
Month +7	Postoperative 4-month follow-up: continued improvement
Month +9	Postoperative 6-month follow-up: marked aesthetic improvement
Month +15	Postoperative 12-month/15-month after initial visit: satisfactory outcome in operated eye; substantial regression in unoperated eye

PPE, personal protective equipment; AMT, amniotic membrane transplantation.

During the follow-up examination three months later, we observed a partial reduction in the patient‘s conjunctival pigmentation compared to the initial assessment (Figure 1). Given the patient's strong desire for further cosmetic improvement, we recommended and performed selective excision of the pigmented lesion in the left eye to clarify the pathological nature and address aesthetic concerns. During the procedure, we selectively removed the severely pigmented areas on the nasal and temporal sides of the conjunctiva using square-shaped incisions with complete dissection, preserving the tissue medial to the limbus to protect limbal stem cells, followed by amniotic membrane transplantation (AMT) using 10-0 nylon interrupted sutures fixed to the superficial sclera to effectively promote conjunctival regeneration and repair in the excised regions (Figure 2). The left eye was selected for surgical intervention, while the right eye was followed as a non-operated contralateral eye (untreated fellow eye). Postoperatively, the patient received levofloxacin and dexamethasone eye drops four times daily; sutures were removed one week after surgery. No intraoperative or postoperative complications occurred. Pathological examination of the resected tissue revealed a moderate increase in melanocytes within the conjunctival epithelial basal layer, without cellular atypia, nesting, pagetoid spread, pigment-laden macrophages, or foreign material (Figure 2). The patient expressed his perspective as follows: “I was very concerned about the appearance of my eyes and felt self-conscious in social situations. After the surgery, I am much more satisfied with how my eyes look.”

**Figure 2:**
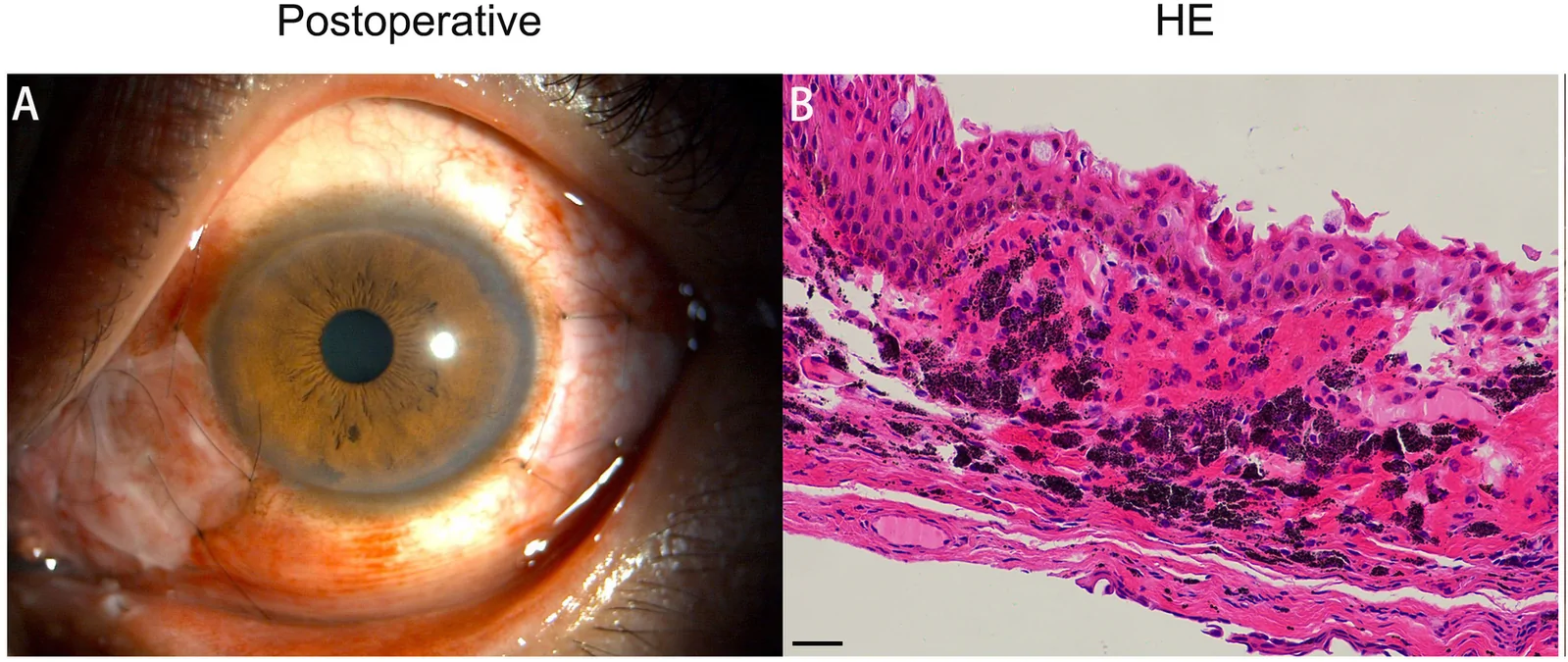
Seven days after left conjunctivectomy combined with amniotic membrane transplantation. **(A)** Slit-lamp photograph showing the amniotic membrane graft in place. **(B)** Left eye histopathology (hematoxylin and eosin, ×200) showing increased melanocytes within the conjunctival epithelial basal layer without atypia (scale ba*r* = 50 μm). No nesting, pagetoid spread, pigment-laden macrophages, or foreign material was identified.

Postoperative follow-up was conducted at 1, 4, 6, and 12 months. At 1 month, gradual replacement of the amniotic membrane graft by healthy conjunctival tissue was observed, with moderate aesthetic improvement in the surgical eye‘s appearance (Figure 1). Continued improvement was noted at 4, 6, and 12 months. Visual acuity remained 20/20 in both eyes throughout follow-up, with no recurrence of pigmentation, no new lesion formation, and no surgical complications. The patient opted for continued observation of the unoperated contralateral right eye (Figure 1), with detailed findings presented in Figure 3. In the unoperated right eye, after 1 year of continued avoidance of exposure (i.e., approximately 15 months from the initial visit), the pigmentation had further decreased substantially (Figure 1). Long-term follow-up at 6 and 12 months showed continued improvement in the operated left eye and persistent reduction of pigmentation in the untreated right eye.

**Figure 3:**
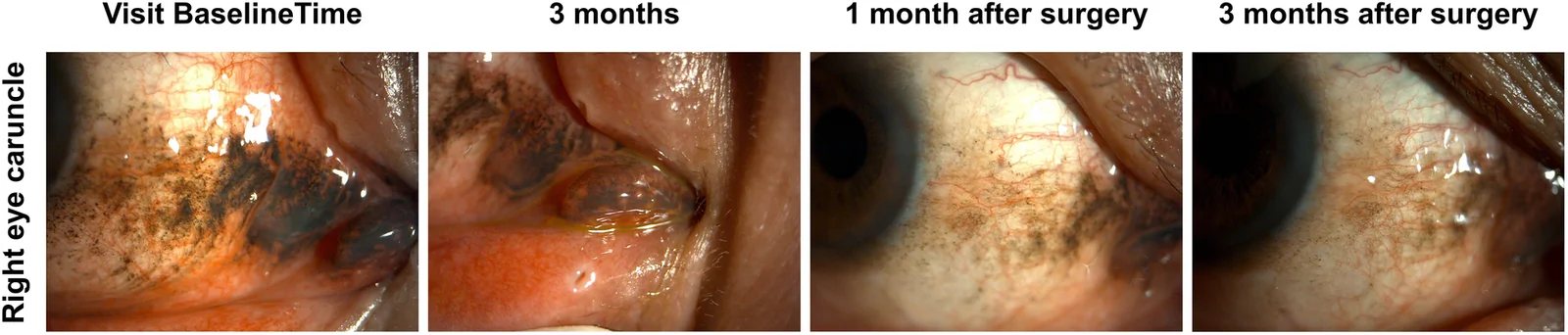
Serial slit-lamp photographs of the untreated fellow eye (right eye) showing gradual regression of pigmentation in the nasal conjunctiva and lacrimal caruncle over time, from baseline, 3 months after exposure cessation, and 1 and 3 months after left eye surgery (i.e., 4 and 6 months after initial visit).

## Discussion

Conjunctival pigmentation is a common ophthalmic condition that can be caused by endogenous and exogenous factors. Endogenous factors include genetic predisposition and hormonal imbalances, while exogenous factors include ultraviolet radiation, trauma, foreign bodies, and exposure to environmental pollutants (1, 2). Organic compound pollution is a prevalent issue in modern industrial production. Phenylenediamine is commonly used in hair dyes and as an intermediate in dye manufacturing and rubber antioxidants. There is a previous report of a worker in a chemical company that handled phenylenediamine developing pigmented patches on the sole of the right foot, although histopathological examination did not reveal any atypical proliferation of melanocytes. The pigmented patches disappeared after surgical removal of the lesion‘s keratinized layer (5). Other organic compounds may also cause skin and mucosal pigmentation. Phenol, a widely used industrial chemical, has been reported to induce pigmentation in the treatment of vitiligo (6). Hydroquinone (HQ) has long been used as a whitening agent, but it carries the risk of causing irritant dermatitis, potentially resulting in further pigmentation issues (7). In the 1940s, Sterner (8), Oglesby (9), and Anderson (10) extensively described cases of conjunctival and corneal pigmentation caused by HQ exposure. Systemic exposure to phenylenediamine and related aromatic amines may also cause dermatologic reactions including allergic contact dermatitis and respiratory irritation. These reports support the notion that organic compounds may contribute to skin and mucosal pigmentation, although the specific mechanisms require further study.

Most previously reported cases are associated with direct contact exposure or occupational hazards involving high concentrations of chemicals. Several case reports have described conjunctival pigmentation associated with chemical or drug exposure, including long-term use of tetracycline and minocycline, as well as exposure to cosmetic pigments, suggesting that exogenous substances can accumulate in conjunctival tissues and lead to pigmentary changes (11–13). Cases of bilateral, diffuse conjunctival pigmentation induced primarily through occupational airborne exposure without direct ocular contact are rarely described in the literature.

While phenylenediamine is common in industrial applications, cases of significant and widespread melanin deposition in the conjunctiva of both eyes associated with occupational airborne exposure—without direct contact history—remain relatively rare. In the present case, the patient developed bilateral conjunctival pigmentation following prolonged occupational exposure to airborne phenylenediamine without using personal protective equipment. Notably, partial regression of pigmentation was observed after avoidance of further exposure, suggesting a certain degree of reversibility.

The differential diagnosis of bilateral conjunctival pigmentation includes complexion-associated melanosis (CAM), primary acquired melanosis (PAM), conjunctival nevus, melanoma, drug-induced pigmentation, and exogenous pigment deposition. CAM typically presents as diffuse, symmetric, perlimbal pigmentation in individuals with darker skin phototypes; our patient‘s lesions were multifocal and scattered rather than diffuse, and he had no family history of CAM. Drug-induced pigmentation was excluded because the patient had no history of any oral or topical medications prior to the onset of pigmentation. A comprehensive review of drug-induced pigmentation has documented that numerous agents, including antimicrobials, antimalarials, and psychotropic drugs, can cause cutaneous and mucosal hyperpigmentation (15); none of these were used by our patient. PAM was excluded because of the bilateral presentation, rapid onset over three months, spontaneous regression after exposure cessation, and histopathological absence of atypia. Melanoma was ruled out by the absence of cellular atypia, nesting, or pagetoid spread on histology. As noted in a recent comprehensive review, histopathological evidence of cytologic atypia is the cornerstone for diagnosing conjunctival melanoma (14).

Exogenous pigment deposition was considered as the primary hypothesis given the clear occupational exposure history. Notably, exogenous conjunctival pigment deposition from environmental exposure has been reported to masquerade as melanoma (16), underscoring the importance of thorough occupational history taking in patients presenting with bilateral conjunctival pigmentation of unclear etiology.

Accurate identification of the underlying cause is crucial for treating conjunctival pigmentation. Non-steroidal and steroid-class agents do not appear to be effective. If bilateral conjunctival lesions are related to occupational exposure to organic air pollutants and the area of melanin deposition is large, immediate avoidance of further contact with the exposure source is extremely important; even without surgery, pigmentation can be expected to partially or even markedly resolve over time. This case indicates that after avoidance of the causative agent, conjunctival pigmentation can improve spontaneously; surgery may be considered for patients with severe pigmentation and cosmetic concerns. However, it must be emphasized that avoidance of further exposure is the primary and most critical intervention.

Several limitations of this case report should be acknowledged. First, as with most retrospective case reports, pre-exposure ocular photographs were not available; this is a practical limitation inherent to the study design rather than a methodological deficiency. Second, we cannot provide direct tissue-level chemical confirmation that the deposited pigment contains phenylenediamine or its metabolites, as ancillary studies such as mass spectrometry or energy-dispersive x-ray spectroscopy were not performed; this reflects routine clinical practice rather than an oversight, as such analyses are not standard for benign conjunctival pigmentation. Third, environmental exposure concentration data were not available, as retrospective case reports cannot feasibly obtain historical workplace monitoring data. Fourth, we did not perform objective quantitative measurement of pigmentation intensity, relying instead on serial clinical photography. Fifth, as the patient was the sole worker in the storage area, no information regarding similar findings in coworkers could be obtained. Finally, the possibility of individual susceptibility vs. a generalized occupational hazard cannot be determined from a single case.

## Conclusion

Conjunctival pigmentation may be associated with prolonged occupational exposure to airborne organic compounds. Avoidance of the causative agent (removal from the contaminated environment) is the primary management strategy, and the condition has the potential for spontaneous improvement. For patients with severe pigmentation and persistent cosmetic concerns after conservative observation, surgical intervention (conjunctival resection combined with amniotic membrane transplantation) may be considered as an option. This case provides deeper long-term insight into ocular surface pigmentation associated with occupational exposure to airborne organic compounds, and highlights the importance of taking a thorough occupational history in patients presenting with bilateral conjunctival pigmentation of unclear etiology.

## Data Availability

The original contributions presented in the study are included in the article/supplementary material, further inquiries can be directed to the corresponding author/s.
